# Mycophenolate mofetil for scleroderma-related interstitial lung disease: A real world experience

**DOI:** 10.1371/journal.pone.0177107

**Published:** 2017-05-25

**Authors:** Misbah Baqir, Ashima Makol, Thomas G. Osborn, Brian J. Bartholmai, Jay H. Ryu

**Affiliations:** 1 Division of Pulmonary and Critical Care Medicine, Department of Internal Medicine, Mayo Clinic, Rochester, Minnesota, United States of America; 2 Division of Rheumatology, Department of Internal Medicine, Mayo Clinic, Rochester, Minnesota, United States of America; 3 Department of Radiology, Mayo Clinic, Rochester, Minnesota, United States of America; University of Texas Health Science Center at Houston, UNITED STATES

## Abstract

**Background and objective:**

Interstitial lung disease (ILD) remains the number one cause of mortality in scleroderma (SSc). Our goal was to determine the effectiveness of mycophenolate mofetil (MMF) in treating SSc-ILD in a retrospective study.

**Methods:**

A retrospective, computer-assisted search was performed to identify patients with SSc-ILD treated with MMF from 1997 through 2014. We used a novel software tool, Computer-Aided Lung Informatics for Pathology Evaluation and Rating (CALIPER), to quantify parenchymal lung abnormalities on high-resolution computed tomography. Lung function was evaluated at baseline, 6, 12, and 24 months of MMF therapy.

**Results:**

We identified 46 patients (28 females) with SSc-ILD (mean age at diagnosis 55 y) treated with MMF for at least 1 year (majority on 2 gm/day). Twenty-one patients (45.7%) stopped using MMF during the follow up period after the first 12 months, and they took MMF for a median of 2.12 years (range, 0.91–8.93 years). Only 4 discontinued MMF because of disease progression. Compared to baseline, the mean percentage change in forced vital capacity (95% CI) at 6, 12, and 24 months, respectively, was 1.01% (−2.38%-4.39%) (n = 26), 2.06% (−1.09%-5.22%) (n = 31), and −0.07% (−3.31%-3.17%) (n = 30), and the mean percentage change in ILD as measured by CALIPER (95% CI) was −5.40% (−18.62%-7.83%) (n = 18), −1.51% (−14.69%-11.68%) (n = 17), and −8.35% (−20.71%-4.02%) (n = 22).The mean right ventricular systolic pressure (RVSP) remained stable over the study period.

**Conclusions:**

MMF is well tolerated and slows the rate of decline in lung function in SSc-ILD patients, even at doses lower at 3 g/day.

## Introduction

Systemic sclerosis (SSc) can virtually affect any organ system, but the fibrotic and vascular pulmonary manifestations of interstitial lung disease (ILD) and pulmonary arterial hypertension (PAH) remain the leading cause of morbidity and mortality in this autoimmune disease. Multiple studies provide estimates of the prevalence of ILD in SSc, which vary depending on the detection method used. The reported prevalence varies from 53.4% [1] to 90% [2]. The rate of decrease has ranged from 2.6% [3] to 4.2% [4] to as high as 7.1% per year in untreated patients [5]. The incidence of PAH is somewhat lower (20.5%-22.3%) than the incidence of ILD [6], [7], but the median survival of some patients with PAH is 1.5 to 2 years after the diagnosis [8].

The pathogenesis of SSc involves a complex interplay of vascular endothelial injury, inflammation, and fibrosis. The presence of inflammatory cells in the bronchoalveolar lavage from these patients signifies alveolitis and active inflammation, and potentially a benefit from immunosuppressive therapy for SSc-ILD [5].

Glucocorticoids are well known triggers for SSc renal crisis and thus doses over 15 mg are generally not recommended for SSc-ILD. Moreover, there is no evidence to support the efficacy of glucocorticoids in this scenario. Among the immunosuppressants studied, oral cyclophosphamide (CYC) has demonstrated benefit randomized control trials. In Scleroderma Lung Study (SLS) I, 12 months of oral cyclophosphamide (CYC) therapy was associated with a small but significant improvement in pulmonary function besides, dyspnea score, skin sclerosis, and health-related quality of life compared with placebo. In absence of therapy however, beneficial effects waned by 24 months and no significant differences from placebo were identifiable for most outcomes [3]. Besides this, toxicity related concerns (bone marrow suppression, gonadal failure, teratogenicity, and hemorrhagic cystitis) limit a clinician’s comfort in using CYC long term. The Fibrosing Alveolitis in Scleroderma Trial studied intravenous CYC for 6 months followed by azathioprine and found no significant difference in FVC, DLCO, HRCT appearance or dyspnea scores [9]. A meta-analysis of the effects of CYC on pulmonary function also found no significant improvement with treatment [10].

The benefit and relative safety of MMF in SSc-ILD has been evaluated in various small observational studies[11–15] with short follow up periods. Most of these studies lacked data from follow up high-resolution computed tomography (HRCT) assessments that were either not obtained or were obtained only at baseline to establish the diagnosis of ILD. Due to the lack of sustained improvement seen with CYC and the potential benefits observed with MMF, the SLS-II study was conducted and results recently reported. This was an elegantly designed 14 center randomized, double-blind, parallel group trial that compared 2 years of oral MMF therapy (target dose 3 g/day) to oral CYC (2 mg/kg/day) for 1 year followed by placebo. Unfortunately, the trial failed to meet its primary-end point of proving superiority of MMF on lung function over CYC at 24 months, but demonstrated clear improvements in lung function with both agents during the 2 year follow up, and supported the preference for MMF given its better tolerability and side effect profile. The primary aim of our retrospective study was to examine the efficacy of 2 years of MMF therapy for SSc-ILD in real world clinical practice for stabilizing or improving lung function measured as % FVC and DLCO. We have also added HRCT assessments, including the use of Computer-Aided Lung Informatics for Pathology Evaluation and Rating (CALIPER) software (developed at Mayo Clinic) to quantitate features of the lung parenchyma. The secondary aims of this study were to evaluate the efficacy of 2 years of MMF therapy for stabilizing or improving PAH, as measured indirectly by right ventricular systolic pressure (RVSP) on echocardiography. We also gathered data on the side effects reported after the initiation of MMF therapy and what dose of MMF was used.

## Materials and methods

A retrospective, computer-assisted search was performed to identify patients who had SSc-ILD and were treated with MMF for at least 1 year from 1997 through 2014. The tool we used for this query was a software available in our institution called Data Discovery and Query Builder (DDBQ) where we used “scleroderma”, “systemic sclerosis” “Scl” as search terms and generated a list where it was used in clinical notes. We initially came up with around 600 patients; we later selected 116 patients who also had ILD and a correct diagnosis of Scl made by a rheumatologist. Out of these, we came up with 46 patients who used MMF for at least one year and we had enough data about them. Our study was approved by the Mayo Clinic Institutional Review Board. Medical records were accessible only if patients had given written permission to allow their records to be used for research.

### Patients

Patients were eligible if they were at least 18 years old; had SSc-related ILD (with the diagnosis of SSc made by a rheumatologist); and had used MMF for at least 1 year. Patients were excluded if they had mixed connective tissue disease or overlap syndromes; if their medical record did not have results for pulmonary function tests (PFTs) and HRCT; or if the patient did not have follow-up of at least 1 year at our institution. There were 29 additional cases of Scl-ILD who were not included in our study either because we did not have any follow up data after the start of MMF in our records or they stopped using the drug in less than a year. The lack of follow up data was the most common (n = 15, 52%) cause of not including these cases in our present study. The other causes of stopping the drug before one year were gastrointestinal causes like nausea or diarrhea, n = 4, death n = 3, disease getting worse n = 1, blisters on fingers n = 1, thrombocytopenia n = 1, shingles n = 1, skin cancer n = 1, swelling in hands n = 1 and repeated infections n = 1. Of note, 23 (50%) of the patients in this study were treated by other immunosuppressant agents before being on MMF.

### Study measures and assessments

Data on demographics and other variables were collected at the beginning of MMF therapy (baseline) and at 6, 12, and 24 months after beginning MMF therapy. Demographic data included age, sex, race, smoking history, and date of SSc-ILD diagnosis. Dates of initiation and discontinuation of MMF therapy were collected and reason for discontinuation, if therapy was stopped. Side effects reported after the use of MMF and the dose of MMF were also collected. Additional data abstracted included: scleroderma specific antibodies; PFT results at baseline, 6, 12, and 24 months after starting MMF therapy; radiologic interpretation of HRCT scans at baseline and at 6, 12, and 24 months after starting MMF therapy; CALIPER data as percentage ILD, percentage ground-glass opacity (GGO), percentage honeycombing, and percentage reticular opacities at baseline and at 6, 12, and 24 months after starting MMF therapy; and echocardiographic RVSP measurements at baseline and at 6, 12, and 24 months after starting MMF therapy. The cut-off for the diagnosis of pulmonary hypertension was: 1) mean pulmonary artery pressure >25 mmHg when right heart catheterization was performed; 2) RVSP >50 mmHg on echocardiography [16, 17]. Follow-up dates included the date of latest follow-up and, if the patient had died, the date of death.

### Data processing

The lung was segmented with a density-based morphologic approach that resulted in identifying central airways and central vessels. Airways were segmented differently with repetitive 3-dimensional region growing and connected components analysis. Pulmonary vessels were extracted with a special tubular structure enhancement filter. A thoracic radiologist (B.J.B.) manually segmented the anatomical lobes. A 3-dimensional volume erosion technique was used to determine central and peripheral regions. Tracheal tree analysis with inclusion of a spherical region of 5 cm was used to include perihilar regions into the central zone of each lobe.

### Tissue quantification

With the use of volumetric quantification, the following radiologic parenchymal features were identified: emphysema, GGO, reticular abnormalities, and honeycombing. The volumetric sum of GGO, reticular abnormalities, and honeycombing was used to define *total ILD*, which was then divided by the CALIPER segmented total lung parenchymal volume to define *percentage ILD*.

### Statistical analysis

Categorical variables were compared between groups with the Fisher exact test. The Wilcoxon rank sum test was used to compare continuous variables between the groups. *P* values less than .05 were considered statistically significant. Mixed models were constructed to calculate the difference between variables measured over time, and this was expressed with the 95% CI. Mixed models account for missing data by using all available data for each patient.

## Results

A total of 46 patients were identified who had SSc-ILD and were treated with MMF and followed up at our institution for at least 1 year. Of those patients, 28 (61%) were women; the mean (SD) age of the cohort was 50.6 (±11.9) years; and 25 (56%) were never smokers were previous smokers, and 3 were current smokers (Table 1). During the follow-up period after the first year of MMF use, 21, 18 (39%) patients stopped using MMF. Those patients were treated with MMF for a median period of 2.12 years (range, 0.91–8.98 years), and 25 patients were still using MMF at the latest follow-up. Twelve patients (26%) died in this cohort. The median dose of MMF was 1.5 g daily. Majority (42.5%) of the patients were on 2gm daily. This was followed by 1.5 gm and 1 gm daily, 25% in each category.

**Table 1:** Demographic and clinical features of 46 patients with Scleroderma-related interstitial lung disease.

Feature	Value
Female Sex, No. (%)	28 (60.9)
Age, mean (SD), y	50.6 (11.9)
Median (IR), y	52.7 (IR 21.6–74.1)
Race, No. (%)	
Black	2 (4.4)
White	44 (95.7)
Disease duration, Median (IR), y^1^	0.8 (IR 0.0–37.8)
Smoking status, No. (%)	
Former	18 (39.1)
Never	25 (55.6)
Current	3 (6.7)
Serology, No. (%)^2^	
Anticentromere (ACA) antibody	6 (14.0)
Scl-70 antibody	12 (27.9)
Negative ACA or Scl-70	25 (58.1)
Extent of skin involvement	
Diffuse	6 (13)
Limited	40 (87)

^1^Interval between the diagnosis of scleroderma and start of Mycophenolate mofetil

^2^Serology results were not available for 3 patients.

**Fig 1:**
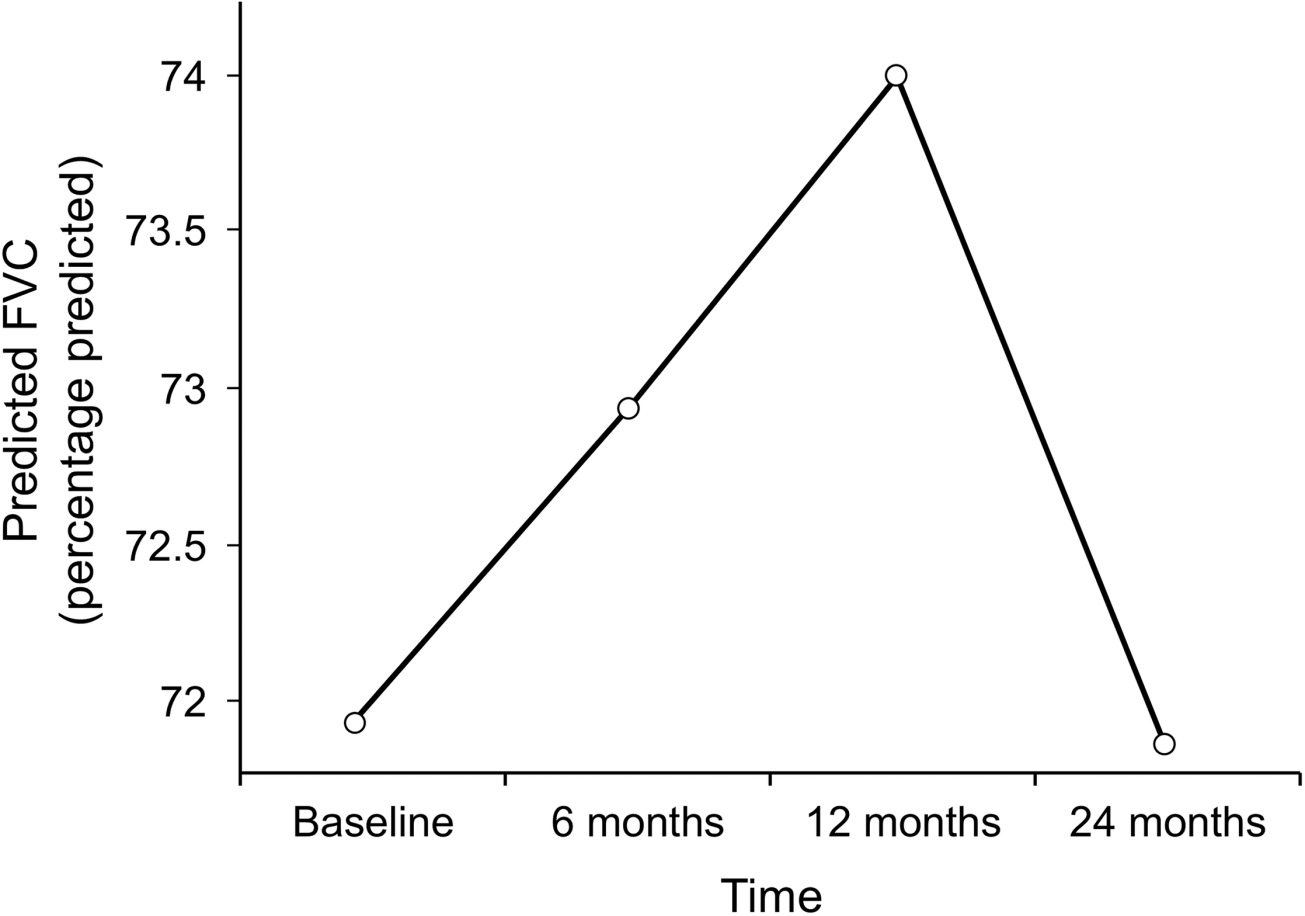
Changes in percentage predicted FVC during mycophenolate mofetil therapy as predicted by mixed model. FVC indicates forced vital capacity.

**Fig 2:**
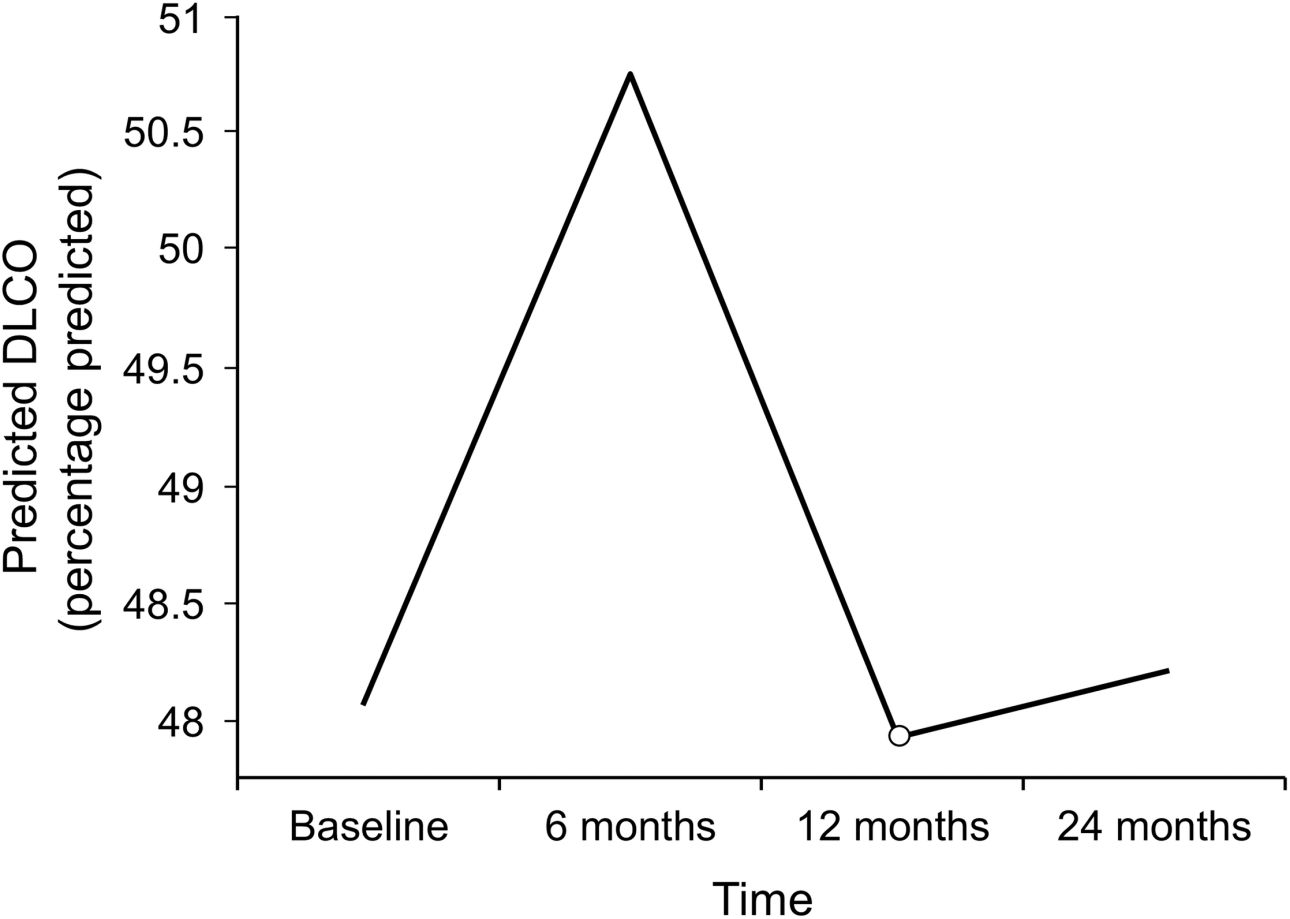
Changes in percentage predicted DLCO During mycophenolate mofetil therapy as predicted by mixed model. DLCO indicates diffusing capacity of lung for carbon monoxide.

PFT, RVSP, and CALIPER data at the start of MMF therapy and at 6, 12, and 24 months after starting MMF therapy are summarized in Table 2. The mean (95% CI) percentage predicted FVC at baseline was 72.1% (66.10%-77.85%). The trend in this change at 6, 12, and 24 months after starting MMF therapy as predicted by our mixed model is shown in Fig 1. The mean percentage diffusing capacity of lung for carbon monoxide (DLCO) (95% CI) at baseline was 48.09% (41.68%-54.50%). The trend in this change as predicted by our mixed model 6, 12, and 24 months after starting MMF therapy is shown in Fig 2. The changes in percentage predicted FVC and in percentage predicted DLCO were not significantly different from the baseline values (Table 2).

**Table 2:** PFT, RVSP, and CALIPER results at baseline and at 6, 12, and 24 months after the start of MMF therapy.

			Change From Baseline								
	Baseline		6 mo			12 mo			24 mo		
Test	Est (95% CI)	n	Est (95% CI)	n	*P*	Est (95% CI)	n	*P*	Est (95% CI)	n	*P*
**PFT**											
FVC, mean % predicted	72.1 (66.10–77.85)	45	1.01 (−2.38–4.39)	26	.56	2.06 (−1.09–5.22)	31	.19	−0.07 (−3.31–3.17)	30	.97
DLCO, mean % predicted	48.09 (41.68–54.50)	45	2.66 (−0.85–6.18)	24	.14	−0.13 (−3.34–3.09)	30	.94	0.14 (−3.08–3.35)	31	.93
**Echocardiography**											
RVSP, mean, mm Hg	41.73 (36.24–47.20)	38	3.03 (−3.59–9.66)	18	.36	−1.97 (−8.3–4.36)	18	.54	−2.36 (−8.66–3.93)	20	.45
**CALIPER**											
ILD, mean %	25.65 (17.14–34.16)	26	−5.40 (−18.62–7.83)	18	.42	−1.51 (−14.69–11.68)	17	.82	−8.35 (−20.71–4.02)	22	.18
GGO, mean %	21.97 (14.38–29.57)	28	−5.24 (−17.64–7.15)	18	.40	−2.99 (−15.38–9.38)	17	.63	−7.60 (−19.21–4.01)	22	.19
Reticular markings, mean %	116.24 (82.25–150.23)	29	−10.05 (−62.28–42.18)	18	.70	28.32 (−25.59–82.24)	17	.29	−18.99 (−70.52–32.54)	22	.46
Honeycombing, mean %	2.32 (−0.44–5.08)	26	0.44 (−3.82–4.70)	18	.84	2.32 (−2.17–6.81)	17	.31	−0.54 (−4.76–3.68)	22	.80

Abbreviations: CALIPER, Computer-Aided Lung Informatics for Pathology Evaluation and Rating; DLCO, diffusing capacity of lung for carbon monoxide; Est, estimated value; FVC, forced vital capacity; GGO, ground-glass opacity; ILD, interstitial lung disease; MMF, mycophenolate mofetil; PFT, pulmonary function test; RVSP, right ventricular systolic pressure.

**Fig 3:**
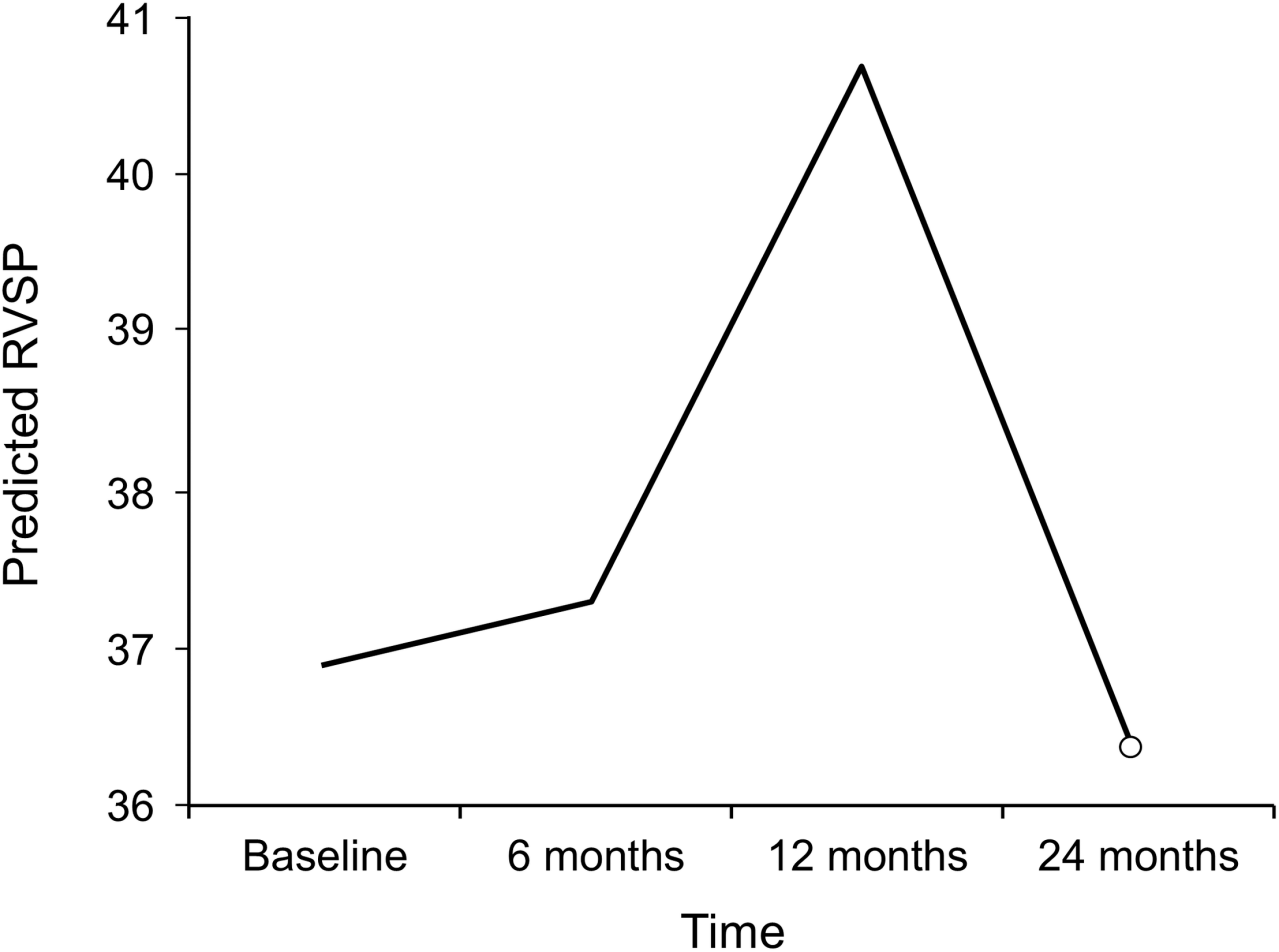
Changes in RVSP during mycophenolate mofetil therapy as predicted by mixed model. RVSP indicates right ventricular systolic pressure.

The most common computed tomographic (CT) chest findings at baseline were nonspecific interstitial pneumonitis followed by GGO (Table 3). The CALIPER data (percentage ILD, percentage GGO, percentage reticular marking, and percentage honeycombing) support the PFT data: There were no significant changes with MMF therapy over time (Table 2). The mean (95% CI) RVSP at baseline was 41.73 mm Hg (36.24–47.20 mm Hg). We found 16 (35%) of the patients to have pulmonary hypertension at the baseline. The trend predicted by our mixed model of this change at 6, 12, and 24 months after starting MMF therapy is shown in Fig 3.

**Table 3:** Disease status on computed tomography of the chest at 6, 12, and 24 months after the start of MMF therapy.

Status	6 mo, % (n = 11)	12 mo, % (n = 15)	24 mo, % (n = 15)
Improved	27	…	…
Stable	36	47	73
Progressed	36	53	27

Abbreviation: MMF, mycophenolate mofetil.

Among the 21 patients who stopped using MMF, 8 died while using it and another 13 patients stopped using it for various reasons. Four people stopped because of disease progression, 1 had increased liver enzymes, 1 underwent lung transplant, 1 had shingles, and 1 had weakness and neurologic symptoms. One patient quit using MMF because his disease was stable. For 4 patients, the reasons for stopping use of MMF were not clear.

Twelve patients in this cohort died, 11 with limited disease and 1 with diffuse. Seven patients had negative serology, 3 were Scl-70 positive and 2 were anti-centromere antibody positive. The causes of death among 8 who died while on MMF were worsening ILD and pulmonary hypertension leading to advanced respiratory failure (n = 4), cancer (lung cancer and spindle cell scalp cancer n = 2), multifactorial with combination of esophagitis, gastrointestinal bleed and ILD(n = 1) and sudden cardiac arrest (n = 1). The causes of death among the other four patients who were not on MMF at the time of death were advanced ILD and pulmonary hypertension (n = 2) and lung cancer (n = 1). In one case the cause of death was not clear.

**Fig 4:**
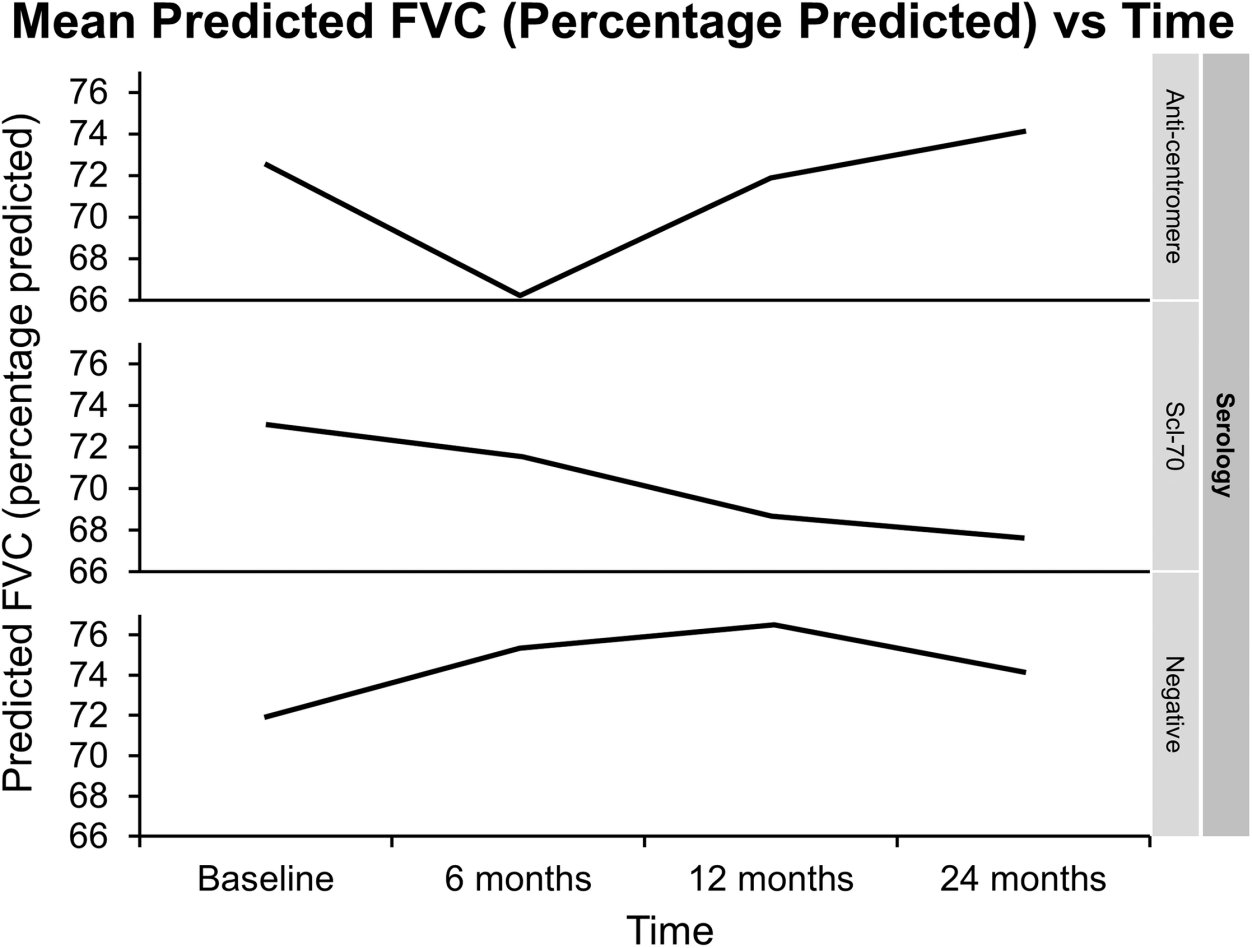
Changes in percentage predicted FVC predicted with mixed model stratified according to the serology of scleroderma.

On exploring whether serology has any relationship with worsening lung function, we did not find any statistically significant effect when serology was added to our mixed model p = 0.10. Although there might be a trend towards lesser decline in FVC (percentage predicted) in anticentromere antibody positive group as shown in the Fig 4. Of note, we only had 6 patients with anti-centromere antibodies. We also tried to see if the extent of skin involvement with Scl has any relationship with decline in FVC and again we did not find any significant difference, p = 0.59. Similarly these two factors (serology and extent of skin involvement) were not found to be significantly affecting the survival, log rank p-values of 0.95 and 0.40 respectively.

Twenty-five patients (20 females, 5 males) were still using MMF at the end of the follow-up period. Twenty patients had limited Scl whereas 5 had diffuse skin involvement; 7 patients had positive Scl-70 antibody, 2 had anti-centromere antibody and 14 had neither of the two antibodies positive. Two patients did not have serology checked.

We gathered data on their latest pulmonary functions and RVSP. The median duration between the latest follow-up and the start of MMF therapy was 3.87 years (range, 2.25–11.5 years). The mean difference in percentage predicted FVC (95% CI) during this time was 0.91% (−2.21%-3.94%) (n = 22, *P* = .54), and the mean difference in percentage predicted DLCO (95% CI) compared to baseline was 1.78% (−1.05%-4.62%) (n = 19, *P* = .20). The mean difference in percentage predicted RVSP (95% CI) during this time was −2.52 mm Hg (−6.04–1.00 mm Hg) (n = 11, *P* = .14). None of these values changed statistically from the start of treatment.

## Discussion

In this retrospective study of 46 SSc-ILD patients, we found that MMF may help to stabilize lung function and pulmonary hypertension for at least 2 years. This drug was generally found to be well tolerated. The PFT results were confirmed by CT of the chest and CALIPER, novel quantitative CT software to evaluate lung parenchyma changes in ILD.

FVC remained stable at 6, 12, and 24 months after the start of MMF treatment. These results are consistent with those from previous studies [11, 15, 18, 19]. Some of the previous studies have shown a significant increase in the percentage predicted FVC after the use of MMF for at least 12 months, but those studies were performed on relatively smaller numbers of patients. The same results were confirmed in large randomized controlled SLS II trial that the patients assigned to MMF experienced improvement in FVC [20]. Our study also found that DLCO was stable during MMF therapy at 6, 12, and 24 months of treatment. These results are consistent with those from most of the previous observational studies related to MMF efficacy and recently published SLS II study.

Changes on CT chest can be subtle over time and difficult to compare, but the use of the CALIPER software supplemented the CT data with quantitative analysis. There were no significant changes in the CALIPER measurements after the start of MMF. Our patients had minimal honeycombing at baseline.

Unlike earlier studies, our study examined the effects of MMF on controlling PAH. We acknowledge the limitations of using RVSP as a measure of pulmonary hypertension. We used echocardiography as it was performed on 38 (83%) of this cohort at baseline whereas right heart catheterization which is the gold standard for the assessment of pulmonary hypertension was performed 18 (39%) of the patients. Moreover even lesser patients underwent right heart catheterization during follow up visits. We found that RVSP was stable while patients took MMF. This could mean that PAH remained stable because the ILD was stable, or it could mean that MMF has a separate role in controlling PAH. Some animal models suggest that MMF is involved in inhibiting the cytokines and vascular smooth muscle receptors, resulting in amelioration of PAH [21]. This finding provides a new insight into the role of MMF in controlling PAH through its antiproliferative effect as an immunosuppressant.

We tried to get more data on the patients, who were still using MMF at the end of the study period, but that was not the primary aim of this study; the information was gathered to have some longer follow-up results. The effects of MMF in stabilizing lung function and RVSP were observed to continue after the 2-year period.

MMF is quite well tolerated; a few patients stopped taking the medication because of its adverse effects. The safety of MMF was confirmed in a meta-analysis of 1 prospective and 4 retrospective studies [22]. This was also reinforced in the recent SLS II study [20].

We acknowledge the many limitations of our study. It was a retrospective observational study with no controls. The stability in lung function during MMF therapy could be due to various factors, including the possibility that the disease may have been in different stages in different patients, or they may have been low-risk patients. Several studies have identified factors affecting the prognosis of the disease, including advanced age [7], usual interstitial pneumonia pattern [23], extent of the lung disease, baseline FVC [24], and duration of the disease [25]. Some of the patients were treated with another agent (e.g., CYC) before beginning MMF therapy. In addition, we excluded patients who used MMF for less than 12 months, might have missed important information about the use of the drug during this initial period.

Our study supports previous studies describing the efficacy of MMF in stabilizing lung function in SSc-ILD. Our data were gathered from a relatively large population and include information not only on PFTs but also on RVSP, CT scans, and quantitative measures for ILD. However, our study does not confirm a role for MMF as induction therapy for SSc-ILD since MMF is generally used as a maintenance treatment only. Our study is a real-life experience outside the boundaries of randomization and supports the safety and tolerability of MMF, and proves that MMF has a beneficial effect in stablising lung function even at doses below those that were found effective in the recently completed SLS II study (2 g vs 3 g) and are often better tolerated in the practical clinical setting.

## Abbreviations

CALIPER: Computer-Aided Lung Informatics for Pathology Evaluation and Rating
CT: computed tomography
CYC: cyclophosphamide
DLCO: diffusing capacity of lung for carbon monoxide
FVC: forced vital capacity
GGO: ground-glass opacity
HRCT: high-resolution computed tomography
ILD: interstitial lung disease
MMF: mycophenolate mofetil
PAH: pulmonary arterial hypertension
PFT: pulmonary function test
RVSP: right ventricular systolic pressure
SSc: scleroderma
